# Novel Immunotherapeutic Approaches for Patients with Head and Neck Cutaneous Squamous Cell Carcinoma

**DOI:** 10.32604/or.2026.069012

**Published:** 2026-03-23

**Authors:** Adam Khorasanchi, Merve Hasanov, Richard Wu, Hisham Alsharif, Kari Kendra, Claire Verschraegen

**Affiliations:** Division of Medical Oncology, Department of Internal Medicine, The Ohio State University Comprehensive Cancer Center, Columbus, OH 43210, USA

**Keywords:** Neoadjuvant, adjuvant, immunotherapy, immune checkpoint inhibitors, head and neck, cutaneous squamous cell carcinoma

## Abstract

Cutaneous squamous cell carcinoma (CSCC) is the second most common type of skin cancer and typically involves the head and neck. Systemic therapy is often required for patients with advanced CSCC to achieve optimal disease control. Immune checkpoint inhibitors (ICIs) are now the standard of care for these patients, with a 50%–60% response rate and sustainable remission for at least 30% of patients. Given the activity of ICIs in advanced head and neck CSCC, ICIs are being studied in early-stage disease or neoadjuvant situations. The purpose of this review is to provide an overview of the innovative perioperative strategies in resectable disease and discuss novel immunotherapeutic strategies designed to overcome treatment resistance. In conclusion, neoadjuvant ICIs have demonstrated impressive response rates with unclear survival benefit. The effectiveness of adjuvant ICIs is currently being explored. Emerging biomarkers, such as circulating tumor DNA (ctDNA), will be crucial for optimizing patient selection and improving treatment outcomes.

## 1 Introduction

In the United States (US), cutaneous squamous cell carcinoma (CSCC) is the second most common non-melanoma skin cancer, with an estimated 1.8 million new cases annually [1]. CSCC predominantly affects older individuals, with a median age at diagnosis between 70–75 years old [2]. Risk factors for CSCC development include prolonged ultraviolet (UV) exposure, lighter skin complexion, the presence of premalignant skin lesions (i.e., actinic keratosis [AK]), and chronic immunosuppression [1,3]. Patients frequently present with scaly, erythematous, or bleeding lesions on sun-exposed areas of the skin. A majority of CSCC cases arise on the head and neck, of which >90% are localized and associated with an excellent prognosis following curative surgery or radiation therapy (RT) [1]. However, in patients with locally advanced or metastatic head and neck CSCC, there is a higher risk for disease recurrence and distant spread, for which systemic therapy is often needed to achieve optimal outcomes [4]. Poor prognostic features of head and neck CSCC include lesions with the following characteristics: (1) large size (>2 cm); (2) significant depth of invasion (>2 mm); (3) poorly differentiated; (4) perineural or lymphovascular involvement (PNI or LVI); and (5) location on the temple, ear, or lip. High-grade and desmoplastic lesions also indicate poor prognosis [5]. Locally advanced head and neck CSCC is defined as tumors with extensive local invasion of deeper tissue structures or multiple disease recurrences following curative intent, for which surgical resection becomes difficult due to the higher likelihood for complications, morbidity, and/or deformity [6]. Additionally, metastatic head and neck CSCC is defined as locoregional (in-transit or regional lymph node metastases) or distant organ disease spread [6].

Before the availability of immune checkpoint inhibitors (ICI), systemic treatment approaches for patients with advanced head and neck CSCC included upfront platinum-based chemotherapy followed by salvage targeted therapy with epidermal growth factor receptor (EGFR) inhibitors. However, these therapies were associated with poor efficacy and lacked durable responses [7]. Furthermore, they caused significant toxicity, which limited their use in older and frailer patients [4]. Immunotherapy produces overall response rates (ORRs) estimated to be 40%–50% in patients with advanced head and neck CSCC [8–10]. Increased efficacy is observed in tumors with higher tumor mutational burden (TMB), perhaps because of increased neoantigen load [8,10,11]. To date, the US Food and Drug Administration (FDA) has approved the ICIs cemiplimab, pembrolizumab, and cosibelimab as first-line treatments for patients with locally advanced or metastatic head and neck CSCC who are not eligible for curative surgery or RT [12–14].

Because immunotherapy is so effective in advanced cutaneous malignancies, there has also been significant interest in using ICIs in the perioperative setting, following the neoadjuvant immunotherapy approach tested for resectable melanoma that led to high pathologic response rates and improved progression-free survival (PFS) [15]. In this review, we further define the role of perioperative immunotherapy in resectable head and neck CSCC. Additionally, we provide guidance on the management of immunotherapy in immunosuppressed patients for which limited prospective data exists. Finally, we discuss novel immunotherapeutic strategies designed to overcome resistance and highlight emerging biomarkers currently under investigation.

## 2 Literature Search Strategy

For this review paper, the search terms “immunotherapy”, “cutaneous squamous cell carcinoma”, “neoadjuvant”, and “adjuvant” were utilized in the PubMed, Google Scholar, and clinicaltrials.gov databases to screen eligible articles. Articles written in English and published from August 2003–August 2025 were included. Articles that did not involve adult human subjects or that were deemed irrelevant to the topic of interest were excluded (Table 1).

**Table 1:** Search methodology

Databases searched	Number of references found	Number of relevant references	Number discarded
**Terms used:** Immunotherapy; Cutaneous Squamous Cell Carcinoma; Neoadjuvant; Adjuvant			**Reasons**: Review articles; not cutaneous squamous cell carcinoma related; not human data
PubMed & Google Scholar	216 articles	140	76
ClinicalTrials.gov	34 trials	17	17
Others (FDA, AJCC, or Industry Notices)	N/A	7	N/A

Note: FDA, Food and Drug Administration; AJCC, American Joint Committee on Cancer; N/A, Not Applicable.

## 3 Head and Neck CSCC Carcinogenesis and Risk Factors

Head and neck CSCC arises from the abnormal proliferation of keratinocytes within the epidermis. It typically develops in a multistep manner, in which there is a progressive accumulation of genetic and epigenetic mutations (i.e., of tumor suppressor genes) following exposure to UV radiation and carcinogens. Common tumor suppressor gene mutations include TP53, NOTCH 1/2, CDKN2A, and RB1, and may result in the transformation of premalignant lesions (i.e., AK, SCC *in situ*) to invasive head and neck CSCC [7,16–18]. Cumulative UV exposure represents a significant risk factor for head and neck CSCC development. UVB radiation can cause direct DNA damage due to pyrimidine dimers, point mutations, and strand breaks, causing genomic instability and the loss of function of tumor suppressor genes [3]. Furthermore, UVA radiation can indirectly cause DNA damage through the formation of free radicals [3]. Chronic immunosuppression is another significant risk factor for head and neck CSCC development, as immunocompetence is essential for cancer surveillance and the elimination of tumor cells [19]. For instance, solid organ transplant recipients (SOTRs) have up to a 100-fold increased risk of cutaneous SCC, with more aggressive behavior and poorer outcomes observed [20]. Other acquired immunodeficiency conditions that place patients at increased risk for head and neck CSCC development include human immunodeficiency virus infection and chronic lymphocytic leukemia. Inherited genetic disorders such as xeroderma pigmentosum cause defective inherent genetic repair mechanisms that confer a higher risk of head and neck CSCC development. Additionally, those with burns, scars, and wounds are at increased risk due to a chronic inflammatory state, which is termed Marjolin ulcers [3]. Finally, several medications, such as antineoplastic agents (fludarabine, hydroxyurea), targeted therapies (BRAF protein inhibitors), biologics (tumor necrosis factor-alpha (TNF-α) inhibitors), and extended use of some antifungals (voriconazole and fluconazole), have been associated with an increased risk for head and neck CSCC development [3,21].

## 4 Head and Neck CSCC Staging and Prognostic Tools

The 2 most common staging methods utilized for head and neck CSCC are the American Joint Committee on Cancer Staging Manual, 8th edition (AJCC8) [22] and the Brigham and Women’s Hospital (BWH) tumor staging system [23,24]. These classification systems are useful for identifying patients at high risk for disease recurrence and metastases, and therefore can guide appropriate treatment strategies [3] (Table 2). The AJCC8 staging system is based on characteristics of the primary tumor, including size, depth and degree of invasion (T), nodal involvement (N), and presence of distant metastasis (M). Tumors with a diameter size < 2 cm (stage T1) or between 2–4 cm (stage T2) are considered low risk. Tumor diameter size > 4 cm, involvement of cortical bone/marrow invasion (stage T3), or skull bone/base invasion (stage T4) are considered high risk [22]. Nodal staging is based on lymph node size, number, and extranodal extension (ENE) [25]. The BWH staging system incorporates several high-risk clinicopathologic factors (tumor diameter size > 2 cm, poor differentiation, PNI ≥ 0.1 mm, tumor penetration beyond subcutaneous fat, or bone invasion) to guide T staging, and has been shown to be a superior prognostic tool for patients with localized head and neck CSCC [24,26]. LVI has not been used but is sometimes documented in pathology reports.

**Table 2:** Comparison of tumor (T) staging in the American Joint Cancer Committee 8th edition (AJCC8) and Brigham-Women’s Hospital (BWH) classification systems

AJCC8 (tumor size, depth of invasion, perineural involvement)		BWH (number of high-risk features*)	
T1	Size < 2 cm	T1	0
T2	Size 2–4 cm	T2a	1
T3	Size ≥ 4 cm; minor bone involvement; perineural spread; deep invasion	T2b	2–3
T4a	Gross cortical bone and/or marrow spread	T3	4
T4b	Skull bone penetration and/or skull base foramen spread		

Note: *High-risk features include tumor diameter ≥ 2 cm, poorly differentiated disease, perineural spread ≥ 0.1 mm, or tumor penetration beyond subcutaneous tissue (excluding bone involvement, which upstages tumor to T3).

In patients with suspected locally advanced or metastatic head and neck CSCC, diagnostic imaging is commonly utilized to evaluate the extent of the primary tumor, PNI on pathology examination, and distant spread (2). Computed tomography (CT) is highly accurate for detecting bony invasion or nodal metastases, while magnetic resonance imaging (MRI) is useful for assessing nerve or deep soft tissue involvement [27]. Additionally, positron emission tomography (PET)/CT imaging may be useful for initial staging and to detect distant metastases [28,29]. If lymph nodes are palpable or detected on imaging, core biopsy may be recommended as regional lymph node involvement confers a significantly increased risk of disease recurrence and mortality [30–32].

Gene expression profile (GEP) testing, which incorporates tumor biology to assess a patient’s risk of developing metastatic disease, is another potential prognostic tool to improve risk stratification of patients with head and neck CSCC. For instance, a 40-GEP test has been developed and validated based on retrospective cohorts integrating tumor biology with clinical features to offer a more nuanced risk stratification compared to conventional staging systems [33]. While this test has not yet been adopted into clinical practice, this GEP-40 test may be useful in guiding which patients may benefit the most from adjuvant RT [34]. Finally, a European web-based prognostic model has been developed, which utilizes 8 readily available clinicopathologic variables to provide an individualized risk assessment of metastatic disease for patients with head and neck CSCC [35]. The Immune Profile Score (IPS) is a validated, multi-omic, pan-solid cancer biomarker using next-generation sequencing (NGS) testing, incorporating both DNA and RNA analysis to predict outcomes of ICI therapy [36]. Such new NGS models could be useful in the future to predict patient outcomes with immunotherapy.

## 5 Treatment Approaches for Head and Neck CSCC

Risk stratification approaches utilize both patient and tumor characteristics to identify patients at high risk for metastases and poor outcomes. According to the National Comprehensive Cancer Network (NCCN) guidelines, all head and neck CSCC lesions are considered high risk regardless of size [32]. Additional high-risk features may include those that are poorly demarcated, rapidly growing, ≥2 mm in depth of invasion, recurrent, present in patients who are being treated with immunosuppressants, located at a site of previous RT or chronic inflammation, expressing specific histologic subtypes (acantholytic, adenosquamous, metaplastic, or desmoplastic), or associated with PNI [32].

### 5.1 Surgery

Surgery represents the mainstay of treatment for patients with high-risk localized head and neck CSCC. Complete surgical margin assessment via Mohs micrographic surgery (MMS) or other peripheral and deep exhaustive margin assessment (PDEMA) techniques is recommended to achieve optimal outcomes [37]. MMS is performed by a trained physician who serves dual roles as both a surgeon and a pathologist. This procedure involves removing thin layers of skin and assessing the presence of cancer cells under microscopy; this process is repeated until clear margins are identified [38]. Advantages of MMS include intraoperative visualization of the entire excision margin and high rates of tissue preservation in cosmetically and functionally sensitive locations. MMS has been associated with improved outcomes, such as lower rates of local recurrence, nodal and distant metastasis, and disease-specific mortality compared to standard excision techniques [39,40]. Other PDEMA approaches differ from MMS in that they involve a team-based approach, in which the procedure is performed by a trained surgeon and the slides are reviewed by a pathologist. Like MMS, these PDEMA approaches have also been shown to have superior outcomes compared to standard excision when there is effective communication among the members of the multidisciplinary team and clear margins are obtained [41]. PDEMA approaches (i.e., Tubingen methods) are preferred over MMS when advanced lesions require excision under general anesthesia due to significant tumor size and/or depth of involvement, or when complex reconstruction is anticipated following removal [42].

A re-excision is recommended for positive margins following surgery. In cases where re-excision is not possible, then adjuvant RT or systemic therapy with immunotherapy is recommended. If negative margins are present following surgery, but extensive PNI or other high-risk features exist, then adjuvant RT is recommended. In the absence of high-risk features, when negative margins are obtained, continued surveillance is recommended, typically by dermatology (every 3–6 months for the first 2 years, every 6–12 months for the next 3 years, and then annually for life) [32].

### 5.2 Radiation Therapy

Definitive RT may be indicated for patients who are poor surgical or immunotherapy candidates because of comorbidities or the extent of disease [32]. Additionally, RT may be indicated if resection would not achieve optimal disease control or if surgery could cause undesirable functional or cosmetic outcomes [43]. Previous studies have demonstrated widely varying recurrence rates ranging from 2.8%–42% following definitive RT, perhaps owing to various techniques or indications, with higher recurrence rates reported in T3/T4 tumors and immunosuppressed patients [44,45].

Adjuvant RT reduces the risk of disease recurrence and is typically indicated for patients with positive margins after surgery, lesions that cannot be fully re-excised, and tumors with PNI or other high-risk features [32]. In patients with positive margins following surgery, adjuvant RT has been shown to improve locoregional control and survival outcomes [46–48]. In patients with negative margins following surgery, adjuvant RT has demonstrated approximately a 50% reduction in local and nodal recurrence [49]. While RT is generally well tolerated, it is toxic. RT-related adverse effects can be classified into acute (≤6 months) or delayed (>6 months). Acute toxicities can persist for several weeks and range from mild erythema to desquamation and necrosis of skin. Delayed toxicities often manifest months or years after RT and may include fibrosis, skin atrophy, lymphedema, skin pigmentation changes, non-healing ulcerations, soft tissue or bone necrosis, or secondary cancers [50]. Due to the increased risk of complications, RT is not recommended for recurrent lesions in a previous radiation field. RT is contraindicated in patients with genetic conditions predisposing them to RT-induced skin cancer, such as nevoid basal cell carcinoma syndrome or DNA repair disorders such as xeroderma pigmentosum and Bloom syndrome. RT is relatively contraindicated in connective tissue diseases such as lupus or scleroderma [32].

### 5.3 Chemotherapy

There are no standardized chemotherapy regimens for the treatment of locally advanced or metastatic head and neck CSCC. Previously utilized cytotoxic chemotherapies include platinum (cisplatin, carboplatin), 5-fluorouracil, methotrexate, bleomycin, doxorubicin, and taxanes (paclitaxel, docetaxel). However, evidence regarding their efficacy is limited to single-arm studies and case series with modest response rates, short durations of response, and significant toxicity. Additionally, chemotherapy represents a major therapeutic challenge in older and frail patients [51,52]. A systematic review of patients with metastatic CSCC treated with cisplatin demonstrated an ORR of 45% with a median disease-free survival (DFS) of 14.6 months [53]. Given its limited efficacy and the increased risk for toxicity, the use of chemotherapy is recommended for patients with locally advanced or metastatic head and neck CSCC who are not candidates for curative surgery or RT and who are ineligible for immunotherapy or experience disease progression following ICI therapy [32].

### 5.4 Targeted Therapy

EGFR inhibitors were one of the first systemic targeted therapies utilized in head and neck CSCC. The EGFR protein is a transmembrane protein, part of the receptor tyrosine kinase (RTK) family. When overexpressed, downstream signaling pathways such as Extracellular signal-related kinase (ERK) and Phosphoinositide 3-kinase/protein kinase B (PI3K/AKT) are activated, which promote cell survival [54–56]. EGFR overexpression is present in 43%–73% of head and neck CSCC cases and may be associated with worse outcomes [16]. EGFR inhibitors can be broadly classified based on their mechanism of action: monoclonal antibodies, which target the extracellular ligand-binding domain of RTK, or small tyrosine kinase inhibitors (TKIs), which inhibit the intracellular tyrosine kinase domain of RTK [57].

Cetuximab, a chimeric (mouse/human) monoclonal antibody, is the most well-studied EGFR inhibitor in head and neck CSCC. Its efficacy was evaluated in a phase 2 trial (NCT00240682) of 36 patients with CSCC. Patients were treated with an initial dose of 400 mg/m^2^, followed by 250 mg/m^2^ weekly for a minimum of 6 weeks. At a 48-week follow-up, the ORR was 28% (8 partial responses [PR] and 2 complete responses [CR]) [58]. Furthermore, a large retrospective study evaluated 58 chemotherapy-naïve patients with CSCC treated with cetuximab monotherapy and demonstrated an ORR of 42% and a disease control rate (DCR) of 70% at 12 weeks [59]. Panitumumab, a humanized monoclonal antibody, was evaluated in a phase 2 trial of 16 patients with advanced CSCC. Patients received a minimum of 3 cycles (6 mg/kg), and a 31% ORR was observed (3 PR and 2 CR) [60]. Dacomitinib is a second-generation small-molecule EGFR inhibitor (human epidermal growth factor receptor (HER) 2/4), which has previously demonstrated efficacy in head and neck SCC cell lines compared to cetuximab [61]. A phase 2 trial (NCT02268747) evaluated the use of dacomitinib in patients with recurrent or metastatic CSCC. The ORR was 28% (26% PR, 2% CR), and the DCR was 86% [62]. EGFR inhibitors are currently recommended for patients who are not candidates for ICIs or who experience disease progression despite ICI treatment [32].

Despite improved efficacy compared to chemotherapy, EGFR inhibitors are limited by their short duration of response (DOR) due to acquired resistance. Mechanisms of acquired resistance include: (1) point mutations in the EGFR protein, altering the ability of inhibitors to bind to their target (i.e., an S492R mutation, which interferes with cetuximab binding); (2) activation of alternative signaling pathways such as amplification of HER2 and Mesenchymal-epithelial transition factor (MET), and AXL receptor tyrosine kinase (AXL) overexpression; (3) mutations in downstream effectors KRAS, BRAF, and PI3K, leading to increased EGFR-independent signaling; and (4) immune evasion by downregulation of major histocompatibility complex-I (MHC-I) or increased programmed cell death ligand 1 (PD-L1) expression [63–65]. Strategies to overcome EGFR inhibitor resistance have included utilizing a synergistic approach by combining EGFR inhibitors with other standard therapies, such as chemotherapy or RT, to improve outcomes [66].

## 6 Immunotherapy

Immunosuppressed patients are at high risk of developing head and neck CSCC, thus highlighting the critical importance of the immune system in cancer surveillance [67]. Checkpoint molecules, such as programmed cell death protein 1 (PD-1) or cytotoxic lymphocyte antigen-4 (CTLA-4), are expressed on the surface of immune cells and their function is to inhibit T-cell activation when bound to their respective ligands. ICIs serve to block these inhibitory pathways, resulting in an augmented antitumor immune response [68]. Head and neck CSCC has one of the highest TMBs among all solid tumors, a predictive feature of ICI response due to increased neoantigen burden [69,70]. This finding has guided the rationale for using ICIs in the management of head and neck CSCC [71]. PD-1 inhibitors have now been incorporated as the standard of care for head and neck CSCC (Table 3).

**Table 3:** Summary of selected prospective ICI studies in advanced head and neck CSCC

NCT number	ICI agent(s)	Phase	Study cohort(s)	Efficacy	Safety	Ref.
NCT02760498	Cemiplimab	2	Unresectable locally advanced/metastatic CSCC	ORR for mCSCC: 47% (35/75 patients) ORR for LaCSCC: 60% (6/10 patients)	AEs in 15% of mCSCC patients 7% of patients stopped treatment due to AEs	[8]
NCT03565783	Cemiplimab	2	Newly diagnosed or recurrent locoregionally advanced resectable head and neck CSCC (stage III–IVA)	ORR: 30% (6/20 patients), pathologic response (pCR+MPR):70% (11/20 patients)	Any-grade TRAEs in 35% of patients All patients had surgery without delay	[79]
NCT04154943	Cemiplimab	2	Resectable stage II–IV (M0) CSCC	pCR: 51% (40/79 pts), MPR: 13% (10/79 patients)	Any-grade irAEs in 15% of patients ≥grade 3 irAE in 4% of patients	[80]
NCT02964559	Pembrolizumab	2	Unresectable locally advanced/metastatic CSCC	ORR: 54% (6/11 pts), DCR 64% (7/11 patients)	Grade 3 TRAEs in 27% of patients	[81]
NCT03284424	Pembrolizumab	2	Unresectable recurrent/metastatic CSCC	ORR: 34% (36/105 patients), DCR: 52% (55/105 patients)	TRAEs in 67% of patients ≥grade 3 TRAEs in 6% of patients	[10]
NCT02883556	Pembrolizumab	2	Unresectable locally advanced/metastatic CSCC	ORR: 41% (16/39 patients)	TRAEs in 71% of patients ≥grade 3 TRAEs in 7% of patients	[76]
N/A	Pembrolizumab + cetuximab	2	Locally advanced/metastatic CSCC	Cumulative ORR: 63%	Single agent ICI: grade 3–4 TRAEs in 16% of patients Combination treatment: grade 3–4 TRAEs in 35% of patients	[82]
NCT03212404	Cosibelimab	1	Metastatic CSCC	ORR: 47% (37/78 patients)	irAEs in 23% of patients ≥grade 3 irAE in 3% of patients	[78]

Note: ICI, immune checkpoint inhibitor; ref, reference; CSCC, cutaneous squamous cell carcinoma; ORR, overall response rate; AEs, adverse events; pCR, pathologic complete response; MPR, major pathologic response; TRAEs, treatment-related adverse events; DCR, disease control rate; irAEs, immune-related adverse events.

### 6.1 Frontline Management

#### 6.1.1 Cemiplimab

Cemiplimab is a PD-1 inhibitor, which was the first ICI approved by the FDA in 2018 for the first-line treatment of patients with recurrent or metastatic head and neck CSCC not amenable to curative surgery or RT. Its efficacy was demonstrated in 2 pivotal clinical trials. Study 1423 was a phase 1 trial (NCT02383212), which enrolled 26 patients with locally advanced or metastatic CSCC who received intravenous (IV) cemiplimab (3 mg/kg every 2 weeks) for up to 48 weeks and demonstrated a durable response with an ORR of 50% [8]. This treatment was well-tolerated with a discontinuation rate of only 7%.

The confirmatory phase 2 trial EMPOWER-CSCC 1 (Study 1540; NCT02760498) enrolled 432 patients with locally advanced or metastatic CSCC [8,9]. Five cohorts (1–4 and 6) were analyzed based on disease stage and dosing regimen. Cohort 1 included metastatic CSCC patients who received cemiplimab 3 mg/kg every 2 weeks (*n* = 59) for up to 96 weeks; cohort 2 included locally advanced CSCC patients who received cemiplimab 3 mg/kg every 2 weeks (*n* = 78) for up to 96 weeks; cohort 3 included metastatic CSCC patients who received cemiplimab 350 mg every 3 weeks (*n* = 56) for up to 54 weeks; cohort 4 included locally advanced or metastatic CSCC patients who received cemiplimab 600 mg every 4 weeks (*n* = 63) for up to 48 weeks; and cohort 6 included locally advanced or metastatic CSCC patients who received cemiplimab 350 mg every 3 weeks (*n* = 167) for up to 108 weeks. This study demonstrated high ORR rates and durable responses across the treatment arms.

A real-world multicenter study assessed 151 patients with locally advanced or metastatic CSCC who received cemiplimab through the Dutch DRUG Access protocol program, including patients with a history of renal transplant or autoimmune diseases [72]. The ORR was 35.1% and a durable benefit was observed in most patients. Notably, treatment-related adverse events (TRAEs) occurred in approximately 30% of patients, of which kidney transplant rejection (*n* = 4/7 enrolled patients) was the most common TRAE.

A retrospective multicenter study was conducted among 131 patients with locally advanced or metastatic CSCC treated with cemiplimab. The ORR was 58% and DCR was 72%; 9.2% of patients experienced grade 3/4 immune-related adverse events (irAEs) necessitating treatment discontinuation, and 2 deaths were reported [73].

In conclusion, cemiplimab offers a high remission rate of around 60%, but can sometimes cause life-threatening AEs. The pathological CR (pCR) rate ranges from 13%–22% with many patients obtaining a cure [11,74]. However, these patients are still at risk for developing new primary CSCCs and require lifelong monitoring, usually by dermatology.

#### 6.1.2 Pembrolizumab

Pembrolizumab is a PD-1 inhibitor, which was approved by the FDA in 2020 for the frontline treatment of patients with recurrent or metastatic head and neck CSCC not eligible for curative surgery or RT. First, KEYNOTE-629 was a multicenter phase 2 trial (NCT03284424), which evaluated the efficacy of IV pembrolizumab (200 mg every 3 weeks, for up to 35 cycles) in 159 patients, including 54 with locally advanced disease and 105 with recurrent or metastatic CSCC [75]. Among those with recurrent/metastatic CSCC, most (87%) had been treated with prior systemic therapy. The ORR for patients with recurrent/metastatic CSCC was 35%, of which 10.5% achieved CR and 78% had a DOR ≥ 12 months. Pembrolizumab was well-tolerated with no new safety concerns reported.

The investigator-initiated phase 2 CARSKIN trial (NCT02883556) evaluated the use of first-line pembrolizumab (IV 200 mg every 3 weeks, for up to 2 years) in 57 patients with advanced CSCC without prior systemic therapy exposure [76]. The ORR improved to 42%, observed at 15 weeks, with a significantly higher ORR (55%) reported in patients with PD-L1-positive tumors compared to tumors without PD-L1 expression (17%). None of the patients experienced disease recurrence within the study follow-up period (22.4 months); however, 1 patient was found to have a new primary head and neck CSCC. Additionally, the median PFS was 29 weeks (95% confidence interval [CI]: 15 weeks-not reached [NR]) and median overall survival (OS) was 108 weeks (95% CI: 61 weeks-NR).

A real-world retrospective study of PD-1 inhibitors, including pembrolizumab, was conducted in advanced CSCC patients across 6 skin cancer centers in Germany [77]. Among 46 evaluable patients, an ORR of 58.7% and DCR of 80.4% were observed. Additionally, the estimated PFS rates at 1 and 2 years were 59% and 52%, respectively.

#### 6.1.3 Cosibelimab

Cosibelimab is a PD-L1 inhibitor, which received FDA approval in 2024 for the upfront treatment of patients with recurrent or metastatic head and neck CSCC who are not eligible for curative surgery or RT. Its efficacy was demonstrated in the phase 1 CK-301-101 trial (NCT03212404), which enrolled 78 patients with metastatic CSCC (mCSCC) [78]. Patients were treated with IV cosibelimab (800 mg every 2 weeks). The ORR was 48%, with 54% of patients experiencing a DOR > 12 months. No treatment-related deaths were reported, though 10.3% of patients experienced grade 3 TRAEs.

#### 6.1.4 Other ICIs

Nivolumab, a PD-1 inhibitor, was tested as a first-line systemic therapy for patients with advanced head and neck CSCC not amenable to curative surgery or RT [32]. The efficacy and safety of nivolumab were evaluated in the phase 2 trial (NCT03834233) of 24 patients with advanced head and neck CSCC [83]. The best ORR was 58.3% with no CRs observed. The estimated median PFS and OS were 12.7 months and 20.7 months, respectively. Twenty-five percent of patients experienced ≥grade 3 TRAEs, and 1 patient discontinued treatment due to toxicity.

Ipilimumab, a CTLA-4 inhibitor, is currently approved for use in patients with metastatic melanoma based upon significantly prolonged OS and PFS data in phase 3 studies [84]. Currently, no prospective studies have evaluated its use in head and neck CSCC, and its efficacy is limited to case studies. A case report by Day et al. demonstrated a rapid, durable clinical response in a patient with refractory head and neck CSCC following 4 cycles of ipilimumab [85]. Additionally, our institution has treated 11 patients with refractory head and neck CSCC, including a patient with a renal transplant, with a significant treatment response and durable survival observed in about 40% of cases (unpublished data).

### 6.2 ICIs in the Neoadjuvant Setting

Given the demonstrated effectiveness of ICIs in the treatment of patients with advanced head and neck CSCC, there is now significant interest in their use in the neoadjuvant setting to improve outcomes. A potential benefit of a neoadjuvant ICI treatment approach may include a more robust immune response due to a tumor-*in-situ*, leading to enhanced T-cell expansion [86,87]. Additionally, there is the potential for reduction of tumor burden and improved resectability of tumors in anatomically and cosmetically sensitive locations. Possible disadvantages of neoadjuvant ICI treatment include a missed opportunity for curative intent with surgical resection in non-responders and the potential risk of irAEs, which may further delay surgery [86]. Of note, the rate of recurrence of very advanced head and neck CSCC after surgery could be as high as 30%, encompassing local, regional, and distant sites. There are no known biomarkers to direct treatment selection.

Neoadjuvant cemiplimab was evaluated in a phase 2 trial (NCT03565783) of patients with resectable stage III–IVa CSCC [79]. Twenty patients were treated with 2 cycles of IV cemiplimab (350 mg every 2 weeks) prior to surgery. Eleven patients were found to have a pCR and 3 patients had a major pathologic response (MPR). pCR is defined as the absence of residual tumor following neoadjuvant treatment and is often used as a surrogate endpoint in clinical trials [88]. MPR is defined as ≤10% residual tumor following neoadjuvant treatment [89]. Subsequently, a phase 2 study (NCT04154943) evaluated the use of up to 4 doses of IV cemiplimab (350 mg every 3 weeks) in 79 patients with resectable stage II–IV CSCC prior to surgery [80]. Notably, 51% of patients achieved pCR, and most patients (68%) who had a PR on imaging were found to have a pCR, suggestive of neoadjuvant cemiplimab’s therapeutic efficacy. In an extended follow-up analysis (median follow-up of 18.7 months), none of the 10 patients who achieved pCR were found to have disease recurrence at data cutoff. The estimated median event-free survival (EFS) rates at 12 and 24 months were 89% and 84%, respectively [90].

In conclusion, neoadjuvant cemiplimab demonstrates promising pathologic responses in head and neck CSCC. Further randomized phase 3 trials are accruing, such as NRG-HN014 (NCT06568172), to demonstrate improved OS prior to widespread adoption.

### 6.3 ICIs in the Adjuvant Setting

The rationale for an adjuvant ICI approach is to eliminate micrometastatic disease following curative treatment to improve survival outcomes. Several prospective trials have demonstrated the effectiveness of adjuvant ICIs in the treatment of resectable stage III/IV melanoma at high risk for recurrence, which has prompted interest in their use in head and neck CSCC [91–93]. One potential disadvantage of adjuvant ICI treatment is the risk for irAEs, some of which may be irreversible and contribute to increased morbidity and mortality [94].

The phase 3 KEYNOTE-630 trial (NCT03833167) had planned to investigate the use of adjuvant IV pembrolizumab (up to 9 cycles) in patients with high-risk, locally advanced CSCC; however, this trial was prematurely discontinued due to futility [95,96]. Additionally, the phase 3 C-POST trial (NCT03969004) investigated the use of adjuvant IV cemiplimab (up to 48 weeks) in 415 patients with high-risk locally advanced CSCC [97]. This study demonstrated significantly improved DFS with a 68% reduction in risk of disease recurrence or death following cemiplimab treatment compared to placebo (hazard ratio [HR]: 0.32; 95% CI: 0.20–0.51; *p* < 0.001) [98]. An OS benefit has not yet been demonstrated, although follow-up is ongoing. Further data analyses are needed prior to recommending adjuvant ICIs.

In conclusion, preliminary data regarding the use of adjuvant cemiplimab appear promising, but mature data from the C-POST trial are needed to assess whether there is a survival advantage.

## 7 Challenges and Limitations of ICIs in Head and Neck CSCC

### 7.1 Immunotherapy Resistance Mechanisms in Head and Neck CSCC (Fig. 1)

Although ICIs have significantly improved outcomes in patients with head and neck CSCC, primary resistance remains a major therapeutic challenge, with approximately 40% of patients failing to respond to initial treatment.

**Figure 1:**
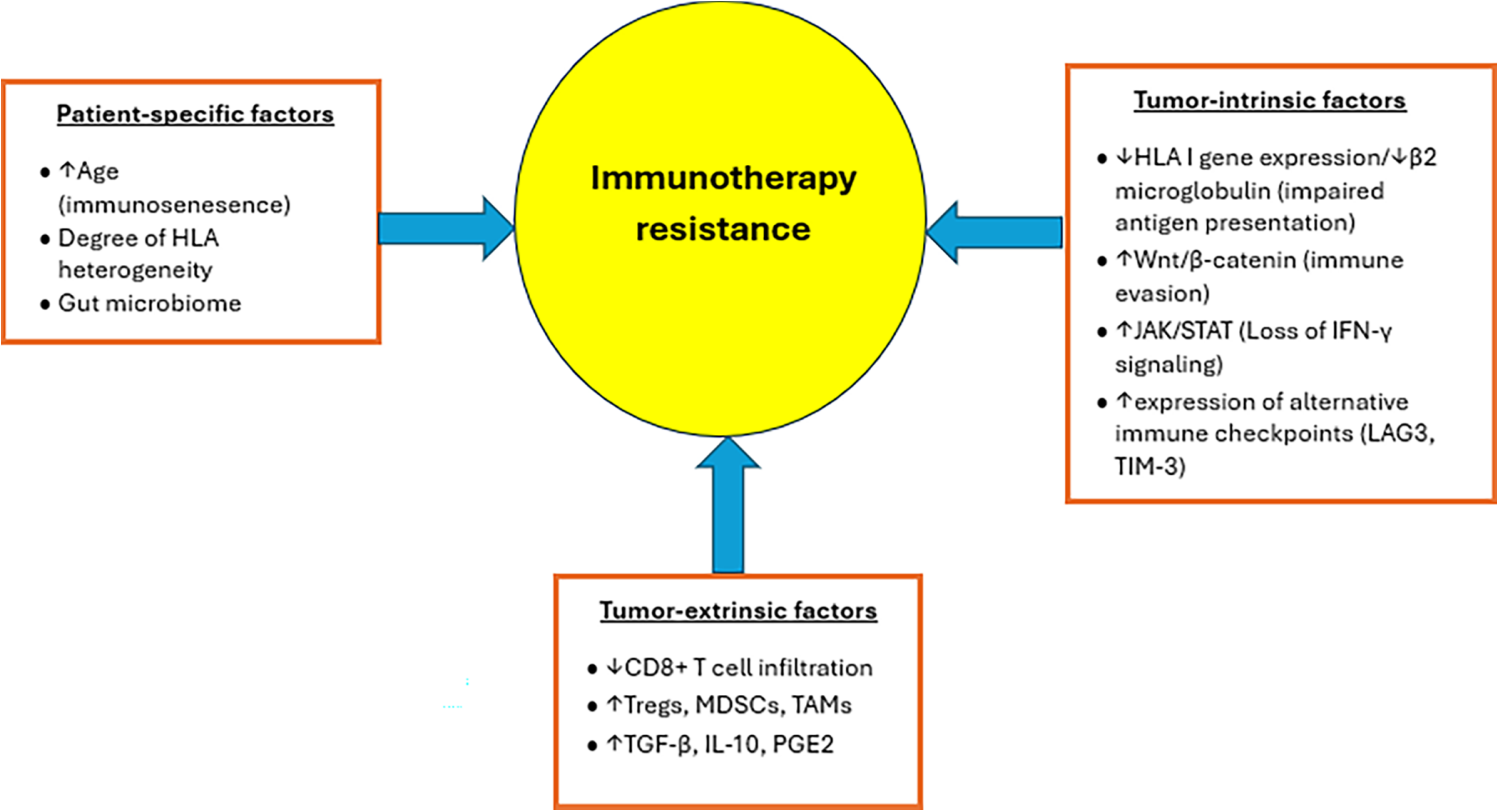
Patient-specific, tumor-intrinsic, and tumor-extrinsic factors contributing to immunotherapy resistance in head and neck CSCC. ↑ denotes increase, while ↓ denotes decrease

Additionally, even among responders, acquired resistance can develop over time, further contributing to lower response rates [99]. Possible reasons for primary ICI resistance may include immunosenescence, which is common in older individuals and results in impaired ICI efficacy due to increased T-cell exhaustion, and a higher proportion of immunosuppressive cells within the tumor microenvironment (TME) [100–102]. The gut microbiome has also been implicated in modulating ICI responsiveness [103,104]. Tumor-intrinsic factors responsible for ICI resistance may include downregulation of human leukocyte antigen (HLA) expression and upregulation of alternative signaling pathways [105]. For instance, abnormal activation of the Wnt/β-catenin signaling pathway leads to decreased chemokine production, which results in impaired T-cell recruitment and infiltration, thus favoring an immunosuppressive TME [106]. Additionally, mutations in Janus kinase (JAK) signaling have been shown to impede interferon-γ signaling, resulting in impaired antigen presentation and T-cell-mediated cytotoxicity [107]. Tumor-extrinsic factors may include the increased presence of immunosuppressive cells within the TME, which impairs T-cell infiltration [99]. For example, perineural invasion was reported to be a mechanism of immune resistance to anti-PD-1 therapy via IL-6 and type I-interferon secreted by the injured nerve [108].

Finally, mechanisms responsible for acquired ICI resistance may be due to alterations in HLA expression and interferon-γ signaling, as well as impaired recognition of tumor antigen, further contributing to immune evasion [109], as suggested by the susceptibility of chronic lymphocytic leukemia patients to head and neck CSCC [108].

Given the challenges associated with ICI resistance in head and neck CSCC, current research has been directed toward combinatorial treatment strategies (through a synergistic approach) and novel targets to improve outcomes. One potential treatment approach is the use of ICIs in combination with RT. Previous studies have suggested that RT could enhance the effectiveness of ICIs by inducing the release of damage-associated molecular patterns (DAMPs) from tumor cells, leading to an augmented antitumor immune response via enhanced antigen presentation and T-cell priming [110]. The use of avelumab in combination with RT in patients with unresectable CSCC is currently being evaluated in the UNSCARRed study (NCT03737721) [111]. A second approach is to use EGFR inhibitors in combination with ICIs. EGFR inhibitors may promote a more inflamed TME through increased T-cell infiltration and activity, reduced immunosuppressive signals, and enhanced antigen presentation [82,112]. The single-arm multicenter phase 2 AliCe study (EudraCT 2018-001708-12) evaluated the use of avelumab (anti-PD-L1, 10 mg/kg every 2 weeks) in combination with cetuximab (500 mg/m^2^ every 2 weeks) for up to 1 year in 54 patients with stage III/IV unresectable CSCC. A majority (66%) of patients in this study had received prior chemotherapy or anti-PD-1 treatment. Preliminary results were presented at the 2023 ESMO Congress: the median follow-up was 2.5 years, and the median PFS and OS among patients in the per-protocol cohort (*n* = 37) was 9.2 months and 25.4 months, respectively [113]. Additionally, 20% of patients experienced ≥grade 3 TRAEs, and 2 patients discontinued treatment due to AEs. Lastly, the multicenter phase 2 I-TACKLE trial (NCT03666325) evaluated the addition of cetuximab to pembrolizumab in 43 patients with locally advanced or metastatic CSCC, once these patients progressed on single-agent pembrolizumab [82]. Among patients exposed to pembrolizumab, an ORR of 44% (19/43) was observed. Of the 21 patients with disease recurrence, 8 (38%) responded to cetuximab despite developing primary resistance to pembrolizumab.

### 7.2 Economic and Logistical Considerations

Despite the widespread use and availability of ICIs in the US, there are several economic and logistical barriers that preclude their administration in more resource-limited countries. First, ICIs are associated with significant financial costs, as the price for a 3-week cycle of dual ICI treatment for melanoma can soar to >$40,000 [114]. Second, considerable infrastructure is necessary to administer ICIs. For example, education and training are needed for medical providers; healthcare facilities must possess specialized equipment to screen eligible patients and monitor toxicity; and well-designed protocols need to be implemented to manage toxicity. Additionally, the lack of reliable supply chains further exacerbates the issue of drug availability. Strategies to improve access to ICIs may include re-evaluating dosing strategies to maximize cost-effectiveness, regional or global price negotiation, development of public-private partnerships, government subsidies, and enabling production of generic ICIs locally through arrangements with pharmaceutical companies to minimize disruption of the drug supply [115,116].

## 8 Novel Therapeutic Targets

Oncolytic viruses (OVs) are genetically engineered intralesional therapies that selectively infect cancer cells, causing lysis and the release of tumor antigens and DAMPs. Collectively, they can induce both local and systemic antitumor immune effects [117]. RP1 (vusolimogene oderparepvec) is a modified non-infectious Herpes simplex virus-1 (HSV-1)-based oncolytic immunotherapy expressing human granulocyte macrophage-colony stimulating factor (GM-CSF) and a fusogenic protein Gibbon Ape Leukemia Virus envelope glycoprotein (GALV) R-modulator that increases immunogenic cell death [118]. The phase 1/2 IGNYTE trial (NCT03767348) evaluated the use of RP1 and nivolumab in 67 patients with advanced cutaneous cancers, including CSCC [119]. A CR rate of nearly 50% was observed following combination therapy, and no deaths related to RP1 were reported, but the study did not meet its endpoint. Another RP1 study is accruing patients with organ transplants (NCT04349436) [120].

Talimogene laherparepvec (T-VEC) is another herpes simplex OV that has been investigated in CSCC. T-VEC has previously shown efficacy in melanoma patients, particularly those with skin, lymph node, and soft tissue metastases [121]. Its use has now expanded to other cancers, including CSCC [122,123]. An interim analysis of a phase 2 trial (NCT03714828) of 7 patients with low-risk CSCC treated with T-VEC demonstrated a CR of 100% [124]. Additionally, a phase 2 trial (NCT03714828) evaluated 11 patients with low-to-intermediate-risk CSCC and demonstrated a CR of 91% following treatment with T-VEC [125].

## 9 Special Considerations

As previously stated, immunocompromised individuals are at an increased risk for head and neck CSCC development, and this has been shown to negatively affect survival outcomes. Immunosuppressed patients experience significantly worse locoregional recurrence-free survival (RFS) and PFS at 2 years [126]. In addition, SOTRs appear to be particularly vulnerable, with one study demonstrating higher rates of CSCC, positive surgical margins, and worse survival outcomes compared to immunocompetent patients [127].

Despite the significant risk of morbidity and mortality in SOTRs, these patients have historically been excluded from head and neck CSCC clinical trials due to the risk of graft rejection following ICI-induced immune activation [128]. Currently, limited data exist regarding the efficacy of ICIs in SOTRs with head and neck CSCC. Therefore, a risk-benefit discussion is recommended prior to initiating ICIs in this patient population, given the significant risk for graft failure (up to 40%–50%) [129,130]. A retrospective study of 133 patients with CSCC (including 10 SOTRs) treated with PD-1 inhibitors demonstrated an ORR and DCR of 80%, though the rate of acute graft rejection was high (20%) [131]. Additionally, a phase 1/2 trial (NCT05896839) evaluated the use of nivolumab, tacrolimus (calcineurin inhibitor), and low-dose prednisone +/− ipilimumab in kidney transplant recipients (KTRs) with advanced cutaneous cancers. Among 8 evaluable patients, none met the primary endpoint (PR, CR, or stable disease at 16 weeks without allograft loss) [132]. There is emerging evidence that the choice of immunosuppressive therapy can mitigate the likelihood of graft rejection in KTRs without compromising treatment efficacy [130]. For instance, the phase 1 CONTRAC-1 study (NCT04339062) evaluated the use of cemiplimab in KTRs with advanced CSCC. Patients received an mTOR inhibitor (sirolimus, everolimus) and pulse-dosed prednisone for immunosuppression. No allograft loss or rejection was reported among 12 patients, and an ORR of 46% (5/11) was observed [133]. One potential approach for the management of immunosuppressed patients involves a multidisciplinary approach to assess whether patients are good surgical and/or radiation candidates. If patients require systemic therapy yet cannot modify their immunosuppressants or are not eligible for clinical trials, a risk-benefit discussion is recommended prior to initiating ICIs [134]. Additional studies are currently underway to further assess the safety and effectiveness of ICIs in SOTR recipients. A phase 1/2 study (NCT05896839) is investigating the use of nivolumab and ipilimumab with prednisone and sirolimus in KTRs with advanced cutaneous malignancies [135]. A phase 1b/2 trial (NCT04349436) is evaluating the use of RP1 in SOTRs with advanced cutaneous malignancies [120]. Responses have been observed (author communication).

## 10 Ongoing Selected Immunotherapy Trials in Head and Neck CSCC

### 10.1 ICIs for Advanced Head and Neck CSCC (Table 4)

There are 5 clinical trials evaluating the use of ICIs in advanced head and neck CSCC. First, a phase 1 trial (NCT05085496) is evaluating the use of stereotactic body RT (SBRT) and atezolizumab (PD-L1 inhibitor) in patients with borderline resectable or unresectable CSCC [136]. This study is estimated to be completed in 2027.

**Table 4:** Selected immunotherapy clinical trials in patients with advanced head and neck CSCC

NCT number	Study population	Immunotherapy agent	Phase	Est. Pts	Trial title	Primary endpoint(s)	Sponsor
NCT05085496 [136]	Borderline resectable or unresectable CSCC	Atezolizumab	1	12	RT + atezolizumab in locally advanced CSCC	DLT, Incidence of AEs	City of Hope
NCT03737721 [111]	Unresectable CSCC, stage I–IV (M0)	Avelumab	2	20	The UNSCARRed Study: unresectable CSCC treatment with avelumab + radical RT	ORR	AHS Cancer Control Alberta
NCT03944941 [137]	Unresectable locally advanced/metastatic CSCC	Avelumab, cetuximab	2	60	Avelumab +/− cetuximab in patients with advanced CSCC	PFS	Alliance for Clinical Trials in Oncology
NCT04204837 [140]	Locally advanced/metastatic CSCC	Nivolumab	2	61	Nivolumab for treatment of CSCC	ORR	Salzburger Landeskliniken
NCT04050436 [138]	Locally advanced/metastatic CSCC	Cemiplimab, RP1	2	231	Cemiplimab +/− RP1 in treating advanced CSCC	ORR, CR rate	Replimune Inc.
NCT04163952 [142]	Locally advanced/metastatic CSCC	T-VEC	1	5	T-VEC + panitumumab for the treatment of locally advanced/metastatic CSCC	Incidence of AEs, ORR	Rutgers
NCT05896839 [135]	Unresectable or metastatic CSCC	Ipilimumab, nivolumab	1/2	16	Immunotherapy + prednisone + sirolimus for KTRs with unresectable or metastatic CSCC	DCR without transplant rejection	NCI
NCT04349436 [120]	Advanced cutaneous malignancies	RP1	1b/2	65	RP1 in SOTRs with advanced cutaneous malignancies	Incidence of AEs, ORR	Replimune Inc.

Note: NCT, national clinical trial; ICI, immune checkpoint inhibitor; est, estimated; CSCC, cutaneous squamous cell carcinoma; DLT, dose-limiting toxicity; AE, adverse event; ORR, objective response rate; PFS, progression-free survival; CR, complete response; KTRs, kidney transplant recipients; DCR, disease control rate; SOTRs, solid organ transplant recipients; T-VEC, talimogene laherparepvec; OV, oncolytic virus; RT, radiation therapy; RP1, vusolimogene oderparepvec; NCI, national cancer institute.

Next, a phase 2 trial (NCT03737721) had planned to evaluate the use of RT and avelumab in patients with unresectable CSCC [111], however was terminated prematurely due to loss of funding. Third, the phase 2 Alliance A091802 trial (NCT03944941) is evaluating the use of avelumab +/− cetuximab in patients with advanced CSCC [137]. It is estimated to be completed in 2028. Additionally, a phase 2 trial (NCT04050436) is evaluating the use of cemiplimab +/− RP1 in patients with locally advanced or metastatic head and neck CSCC [138]. Initial results of this study were disclosed in a press release, in which combination therapy failed to meet either of the study’s primary endpoints (CR, ORR) [139]. It is estimated to be completed in 2025. Finally, a phase 2 trial (NCT04204837) is evaluating the use of nivolumab +/− relatlimab (anti-LAG-3) in patients with locally advanced or metastatic CSCC [140]. Results from the nivolumab cohort were presented at the 2022 ASCO Annual Meeting; among 29 evaluable patients, the best ORR observed was 65.2% along with a DCR of 66.7% and a median PFS of 10.9 months [141]. It is estimated to be completed in 2027.

### 10.2 Neoadjuvant ICIs (Table 5)

There are 4 ongoing trials evaluating the use of neoadjuvant systemic ICIs in resectable head and neck CSCC. A single-arm phase 2 trial (NCT06288191) is evaluating the use of 2 cycles of neoadjuvant IV nivolumab (480 mg) and relatlimab (80 mg) every 4 weeks in patients with treatment-naïve resectable CSCC [143]. This study is estimated to be completed in 2036.

**Table 5:** Summary of selected ongoing perioperative immunotherapy trials in patients with resectable head and neck CSCC

NCT number	Treatment setting	Immunotherapy agent(s)	Pt selection	Phase	Trial title	Primary endpoint(s)	Sponsor
NCT06288191 [143]	Neoadjuvant	Nivolumab + relatlimab	Treatment-naïve, resectable stage II–IV CSCC	2	Neoadjuvant nivolumab + relatlimab in CSCC	pCR rate	Melanoma Institute Australia
NCT05025813 [144]	Neoadjuvant	Pembrolizumab	Stage II–IV CSCC	2	Neoadjuvant pembrolizumab in CSCC	Pathologic response	Queensland Health
NCT04315701 [145]	Neoadjuvant	Cemiplimab	HR localized CSCC, locally recurrent CSCC, or regionally advanced CSCC	2	Cemiplimab for HR localized, locally recurrent, or regionally advanced CSCC	Pathologic PR	University of Southern California
NCT06014086 [148]	Neoadjuvant	PH-762 (RNAi)	CSCC resectable (≥stage II), CSCC unresectable (≥stage II), mCSCC	1	Intratumoral PH-762 for CSCC	Incidence and severity of AEs	Phio Pharmaceuticals, Inc.
NCT06568172 [146]	Neoadjuvant	Cemiplimab	Invasive CSCC or regional LN mets or ITM	3	Cemiplimab + surgery vs. surgery alone for treating advanced CSCC	EFS	NCI
NCT04428671 [147]	Adjuvant	Cemiplimab	Nodal disease with ECE (at least 1 LN > 20 mm), ITM, T4 lesion, PNI, recurrent CSCC	1	Cemiplimab before and after surgery for the treatment of HR CSCC	Pathologic response rate	Emory
NCT03969004 [97]	Adjuvant	Cemiplimab	Resectable HR CSCC	3	Adjuvant cemiplimab vs. placebo after surgery and RT in pts with HR CSCC	DFS	Regeneron Pharmaceuticals

Note: NCT, national clinical trial; pt, patient; est, estimated; pCR, pathologic complete response; PR, partial response; AEs, adverse events; HR, high-risk; CSCC, cutaneous squamous cell carcinoma; RNAi, RNA interference; mets, metastasis; ITM, in-transit mets; EFS, event-free survival; LN, lymph node; ECE, extracapsular extension; PNI, perineural involvement; DFS, disease-free survival.

Next, the single-arm phase 2 DESQUAMATE trial (NCT05025813) is investigating the use of 4 cycles of IV pembrolizumab (200 mg every 3 weeks) in patients with resectable CSCC, followed by interval restaging, prior to surgery [144]. It is estimated to be completed in 2027.

Additionally, a single-arm phase 2 trial (NCT04315701) is investigating the use of neoadjuvant IV cemiplimab (up to 3 cycles) in patients with high-risk localized, locally recurrent, or resectable locally advanced CSCC. Patients may receive an additional treatment cycle if their disease is deemed unresectable after 3 cycles [145]. It is estimated to be completed in 2027. Finally, a phase 3 trial (NCT06568172, NRG-HN014) is evaluating the role of surgery +/− neoadjuvant cemiplimab (up to 4 cycles) in patients with CSCC [146]. It is estimated to be completed in 2031.

### 10.3 Adjuvant ICIs (Table 5)

There are 2 ongoing trials evaluating adjuvant ICIs in resectable head and neck CSCC. First, the phase 3 C-POST trial (NCT03969004) evaluated the use of adjuvant cemiplimab vs. placebo in 415 patients with high-risk resectable CSCC [97]. 209 patients were assigned to the experimental arm and received IV cemiplimab 350 mg every 3 weeks for 12 weeks, followed by cemiplimab 700 mg every 6 weeks for up to 36 weeks. High-risk features in this study were defined as follows: (1) nodal disease with extracapsular extension (ECE) and at least 1 lymph node greater than 20 mm or ≥3 positive lymph nodes noted on surgical pathology, regardless of ECE; (2) in-transit metastases; (3) T4 lesion; (4) PNI; and (5) recurrent CSCC with ≥1 other risk factor [98]. The median follow-up was 2 years, and this study demonstrated improved DFS (HR 0.32, 95% CI 0.20–0.51, *p* < 0.001) along with decreased risk of locoregional and distant recurrence. OS data is not yet mature. The study is estimated to be completed in 2028. Additionally, a phase 1 trial (NCT04428671) is investigating the use of perioperative (neoadjuvant + adjuvant) cemiplimab for the treatment of high-risk localized CSCC. Patients will be treated for up to 3 cycles prior to surgery, and up to 18 cycles after surgery [147]. It is estimated to be completed in 2031.

### 10.4 Intralesional Immunotherapy

There are 3 ongoing trials evaluating intralesional immunotherapy in the management of head and neck CSCC. First, a phase 1 trial (NCT06014086) is evaluating the use of 4 doses of weekly neoadjuvant intralesional PH-762 (anti-PD-1 mRNA) in patients with advanced cutaneous malignancies, including CSCC [148]. The study is estimated to be completed in 2026. Next, a phase 3 trial (NCT06585410) is evaluating the use of intralesional cemiplimab compared to surgery in patients with early-stage CSCC [149]. It is estimated to be completed in 2030. Finally, a phase 1 trial (NCT04163952) is evaluating the use of T-VEC and panitumumab (every 2 weeks, up to 3 cycles) in patients with locally advanced or metastatic head and neck CSCC [142]. It is estimated to be completed in 2025.

## 11 Biomarkers

Currently, there are 3 FDA-approved biomarkers (PD-L1 expression, TMB, microsatellite instability/deficiency of DNA mismatch repair) that are utilized in clinical practice to predict potential ICI response [150]. While higher PD-L1 expression may indicate a superior response to ICI treatment, anti-tumor activity has been observed in CSCC irrespective of PD-L1 expression status [11]. Similarly, patients with higher TMB may derive greater benefit from ICIs; considerable variability in response exists in CSCC and there is no established optimal TMB threshold [80]. For these reasons, PD-L1 expression and TMB status are not used to guide treatment selection for patients with CSCC. There is emerging interest in the development of effective biomarkers that could more reliably predict ICI response and provide useful prognostic information in CSCC [151]. For instance, early immunological changes in IL8 and PD-1+ regulatory T cells within the TME have been shown to predict ICI response and guide treatment monitoring in CSCC tumor samples treated with cemiplimab [152]. Furthermore, expression of other immune checkpoints such as T cell immunoglobulin and ITIM domain (TIGIT) and V-domain Ig suppressor of T cell activation (VISTA) has been shown to correlate with increased CD8+ T-cell infiltration within CSCC tumor samples and may also influence ICI treatment response [153,154].

Circulating tumor DNA (ctDNA), which is the amount of DNA released by tumor cells into the bloodstream, is also emerging as a potential biomarker. Advantages of ctDNA may include its noninvasive nature, the ability to obtain serial measurements to assess tumor burden and treatment response, and the potential to detect minimal residual disease (MRD) and early recurrence, and to predict treatment effectiveness by identifying mutations and potential mechanisms of resistance through comprehensive tumor profiling [155,156]. Additionally, ctDNA positivity has previously been shown to correlate with higher tumor burden and metastatic disease in patients with melanoma [157]. Disadvantages of ctDNA include its cost, availability of testing, lack of standardized assays, unclear optimal threshold value for clinically relevant positivity, inherent limitations associated with detection, and longer turnaround times to obtain results, which can delay clinical decision-making [158]. Moreover, the use of ctDNA assays is currently limited by lower sensitivity rates, particularly in patients with lower tumor burden and early-stage disease. For instance, a retrospective study evaluated the sensitivity of a commercial ctDNA assay (Natera) for the detection of recurrent/relapsed melanoma in patients with no evidence of disease following treatment and an undetectable ctDNA level. This study demonstrated an overall sensitivity rate of 53% and was influenced by the location of disease recurrence/relapse [159]. Additionally, the presence of alterations associated with clonal hematopoiesis of indeterminate potential may result in false-positive findings and further contribute to diagnostic uncertainty [158]. Several studies have investigated the role of ctDNA in patients with CSCC. A single-center study performed ctDNA analysis in 21 patients with intermediate-to-high-risk CSCC previously treated with surgery +/− adjuvant RT [160]. ctDNA was positive in 78% (7/9) of patients with visible disease (either on imaging or physical exam) compared to 11% (1/9) in patients without visible disease and was found to be a more reliable biomarker in patients with higher disease burden. Another study evaluated ctDNA using a PCR assay in a cohort of 36 patients with advanced CSCC [155]. The sensitivity was 63% and patients with regional or distant metastatic disease tended to possess higher ctDNA levels compared to localized disease. Finally, a single-center study evaluated the role of early ctDNA changes and disease response along with survival outcomes in 46 patients with advanced melanoma and other skin cancers treated with ICIs [161]. A decrease in ctDNA levels, 3–4 weeks following treatment, was found to be significantly associated with overall disease control, longer PFS, and better OS. While data regarding ctDNA appear promising, additional prospective studies are needed to further validate these findings. For instance, a trial (NCT06875609) is currently investigating the role of ctDNA in MRD detection and monitoring of immunotherapy treatment response in patients with high-risk CSCC following surgery [145]. In summary, the standardization of ctDNA assays remains critical to improve reliability and reproducibility of test results and better inform clinical decision making [162].

Another emerging biomarker is circulating microRNA (miRNA), which like ctDNA, can be detected in the bloodstream, although it is less tumor-specific [163]. Abnormal miRNA expression has been shown to serve an important role in the formation and progression of CSCC [164]. Thus far, miRNAs have been evaluated as both a diagnostic and prognostic biomarker in CSCC. For instance, a study investigated exosomal miRNA (serum and tissue) in 35 patients with CSCC, and found that higher levels of miRNA-142-5p were associated with later-stage disease and those with disease recurrence [165]. However, several challenges remain prior to the adoption of circulating miRNAs as a validated biomarker in CSCC. These include the need to improve miRNA quantification testing, the adoption of standardized guidelines for its use in clinical settings, and further validation in prospective studies [163].

## 12 Conclusions

ICIs have transformed the treatment landscape for patients with advanced head and neck CSCC with improved efficacy and durable responses compared to previous standard-of-care systemic therapy options. Despite these advances, approximately 50% of patients do not respond to initial ICI therapy, prompting the investigation of innovative immunotherapeutic strategies, including combination regimens and novel molecular targets. Furthermore, there has been a shift toward the use of perioperative ICIs in early-stage disease to improve outcomes. Neoadjuvant ICIs have demonstrated impressive response rates, but whether this translates into a survival benefit is an area of active investigation. Finally, there currently is a paucity of data regarding the effectiveness of adjuvant ICIs, but preliminary results with cemiplimab appear encouraging. An improved understanding of the TME and the emergence of biomarkers such as ctDNA will be necessary to optimize patient selection and maximize treatment outcomes.

## Data Availability

Not applicable.
